# P-2147. Predicting Infection-Related Mortality in Breast Cancer: A Nomogram-Based Risk Stratification Approach

**DOI:** 10.1093/ofid/ofaf695.2310

**Published:** 2026-01-11

**Authors:** Maria Akiki, Rabih Hallit, Chebly Dagher, Souheil Hallit, Ali Hemade

**Affiliations:** University of Connecticut, Hartford, CT; Holy Spirit University of Kaslik, Beirut, Beyrouth, Lebanon; University of Connecticut, Hartford, CT; Holy Spirit University of Kaslik, Beirut, Beyrouth, Lebanon; Lebanese University, Beirut, Beyrouth, Lebanon

## Abstract

**Background:**

Infections are a major cause of illness and death in breast cancer patients. As survival improves, infection risk remains high despite being preventable. This study aims to identify key clinical and demographic factors associated with infectious disease mortality in breast cancer patients and develop a predictive nomogram for individualized risk estimation.

**Figure:**
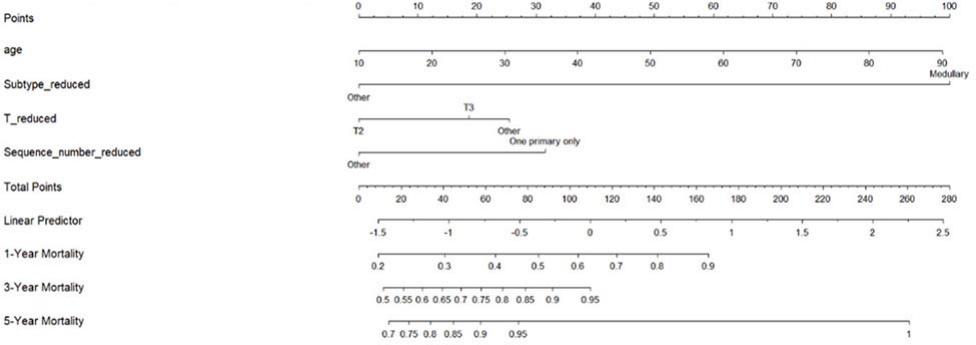
Nomogram predicting risk for death from infectious causes in breast cancer patients. The nomogram estimates 1-, 3-, and 5-year infection-related mortality based on patient age, tumor subtype, tumor stage (T), and whether breast cancer is the only primary cancer. Points assigned to each variable are summed to calculate overall risk.

**Methods:**

We conducted a retrospective cohort study using SEER data from 2010 to 2015. Patients diagnosed with breast cancer were included if they had complete demographic, clinical, and survival data. The primary outcome was infectious disease-related mortality. Key variables included demographic factors, tumor characteristics, and treatment modalities. Kaplan-Meier survival analysis was performed, and Cox proportional hazards regression was used to identify factors associated with infection-related mortality. A nomogram was developed to provide clinicians with a risk prediction tool for infection-related mortality.

**Results:**

Among 43,483 breast cancer patients, 482 died from infectious causes. Older age was a significant predictor of infection-related mortality (HR=1.017, 95% CI: 1.0098–1.0244, p< 0.001). The medullary subtype exhibited an increased risk of infection-related mortality (HR=4.778, 95% CI: 1.3926–16.3955, p=0.0129), while T2 tumor stage was associated with a protective effect (HR=0.7079, 95% CI: 0.5708–0.8779, p=0.0017). Patients with only one primary cancer had a higher risk of infectious disease mortality (HR=1.5737, 95% CI: 1.1841–2.0917, p=0.0018). Patients who received chemotherapy and radiotherapy had improved survival in Kaplan-Meier analysis. A predictive nomogram was constructed with a concordance index of 0.868, demonstrating strong predictive accuracy.

**Conclusion:**

This study identifies key risk factors for infection-related mortality in breast cancer, including older age, medullary subtype, and having a single primary cancer. Chemotherapy and radiotherapy were linked to better survival, likely reflecting patients with better performance status selected for treatment. The developed nomogram estimates individual mortality risk at 1, 3, and 5 years, supporting targeted prevention, reduced infections, and improved outcomes.

**Disclosures:**

All Authors: No reported disclosures

